# Microvascular dysfunction in septic and dengue shock: Pathophysiology and implications for clinical management

**DOI:** 10.21542/gcsp.2020.29

**Published:** 2020-11-30

**Authors:** Angela McBride, Ho Q. Chanh, John F. Fraser, Sophie Yacoub, Nchafatso G. Obonyo

**Affiliations:** 1 Oxford University Clinical Research Unit, Ho Chi Minh City, Viet Nam; 2 Brighton and Sussex Medical School, United Kingdom; 3 Critical Care Research Group, Brisbane, Australia; 4 University of Queensland, Brisbane, Australia; 5 Centre for Tropical Medicine and Global Health, University of Oxford, United Kingdom; 6 KEMRI-Wellcome Trust Research Programme, Kilifi, Kenya; 7 Initiative to Develop African Research Leaders, Kilifi, Kenya

## Abstract

The microcirculation comprising of arterioles, capillaries and post-capillary venules is the terminal vascular network of the systemic circulation. Microvascular homeostasis, comprising of a balance between vasoconstriction, vasodilation and endothelial permeability in healthy states, regulates tissue perfusion. In severe infections, systemic inflammation occurs irrespective of the infecting microorganism(s), resulting in microcirculatory dysregulation and dysfunction, which impairs tissue perfusion and often precedes end-organ failure. The common hallmarks of microvascular dysfunction in both septic shock and dengue shock, are endothelial cell activation, glycocalyx degradation and plasma leak through a disrupted endothelial barrier. Microvascular tone is also impaired by a reduced bioavailability of nitric oxide. In vitro and in vivo studies have however demonstrated that the nature and extent of microvascular dysfunction as well as responses to volume expansion resuscitation differ in these two clinical syndromes. This review compares and contrasts the pathophysiology of microcirculatory dysfunction in septic versus dengue shock and the attendant effects of fluid administration during resuscitation.

## Introduction

The microcirculation is the terminal vascular network of the systemic circulation, whose primary function is to distribute oxygen to, and remove metabolic by-products from living cells. In health, tissue perfusion is regulated by control of microvascular tone and endothelial permeability. In states of shock, however, there is a mismatch between demand for, and delivery or utilisation of oxygen in the tissues^1^.

The microcirculation comprises arterioles, capillaries and post-capillary venules. The primary component of micro-vessels are endothelial cells; arterioles are also surrounded by vascular smooth muscle cells. Endothelial cells maintain the selective vascular barrier by means of dynamic regulation of inter-endothelial adherens and tight junction integrity, as well as control of cytoskeletal contraction by reorganisation of filamentous actin^2^. The luminal surface of vascular endothelium is lined with a fine hair-like structure, the glycocalyx, which forms a physical and electrostatic barrier that is important for vascular homeostasis. The glycocalyx is composed of membrane-bound proteoglycans, sulphated-glycosaminoglycans, as well as glycoproteins bearing acidic oligosaccharides and terminal sialic acids^3^. This elaborate network contains a high density of negatively-charged glycosaminoglycan side-chains that ensure laminar flow is maintained through electrostatic repulsion of intravascular proteins (albumin, globulin, and cellular components) away from the vessel wall towards the centre of the lumen^3^. Other important functions of the glycocalyx include regulating microvascular tone, inhibiting microvascular thrombosis, regulating leucocyte adhesion on the endothelium, and acting as a bio-sensor for as well as mediating mechanotransduction of shear forces exerted by circulating volume^4,5^.

In a normal healthy state, the glycocalyx shields the endothelial cells from oxidative stress, and transmits excess luminal shear forces to endothelial cells thus initiating nitric-oxide mediated vasorelaxation^4^. Vascular homeostasis is also under the control of endothelial-derived prostacyclin, a lipid and endothelin-1, a peptide. Metabolism of arachidonic acid by cyclooxygenase (COX) yields prostaglandin H2, which undergoes further terminal metabolism to produce either prostacyclin (prostaglandin I_2_) or thromboxane A2^6^.

Prostacyclin is a potent vasodilator of both systemic and pulmonary vasculature as well as an inhibitor of platelet activation^7^. It acts via G-protein-coupled receptors to stimulate adenylate cyclase and increase the levels of cyclic AMP which in turn activates protein kinase A (PKA) leading to phosphorylation of myosin light chain kinase and platelet inositol 1,4,5-triphosphate^8^. Thromboxane A2 causes platelet aggregation and vasoconstriction acting synergistically with endothelin-1, a potent endogenous vasoconstrictor secreted by endothelial cells. Endothelin-1 also acts via G-protein-coupled receptors. Endothelin-1 type A receptors are located mostly in vascular smooth muscle cells and are responsible for the vascular contraction, cellular proliferation and proinflammatory effects^9^. Endothelin-1 type B receptors are located on both endothelial and vascular smooth muscle cells and are thought to play a role in clearance of endothelin-1 as well as release of an endothelium-derived hyperpolarizing factor^10,11^. The balance of endothelial vasoconstrictors and vasodilators is important in microvascular homeostasis.

**Figure 1.**
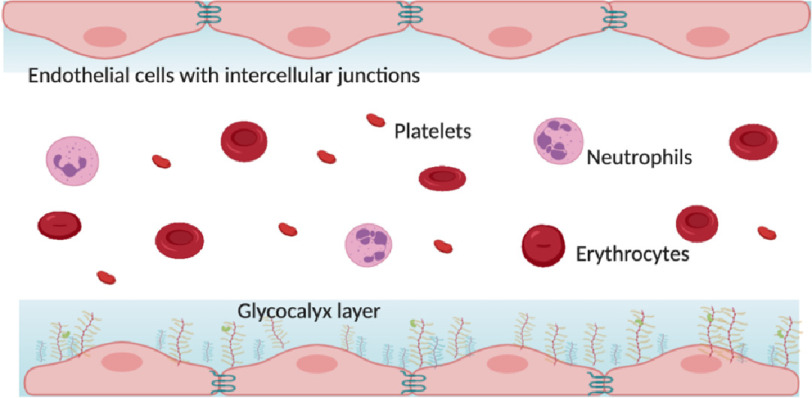
Appearance of the microcirculation under normal conditions with intact endothelial cell junctions and glycocalyx lining.

Figure 1 shows the appearance of the microcirculation under normal conditions

In severe infections, systemic inflammation and direct pathogen-mediated damage can lead to impairment of the microcirculation, which precedes end-organ dysfunction. Progression from severe infection to shock with vasoplegia and multi-organ failure carries a high risk of potentially worse outcomes irrespective of the infecting pathogen^12,13^. The clinical phenotype of patients with shock caused by bacterial sepsis and viral infections like dengue carry some similarities but also are distinct in their presentation and management. This review therefore compares and contrasts the pathophysiology of microcirculatory dysfunction in septic *versus* dengue shock and the attendant effects of fluid administration during resuscitation.

### Sepsis and Dengue

According to the latest iteration of the Global Burden of Disease study, sepsis affects 50 million people worldwide leading to 11 million deaths^14^. Despite advances in the understanding of the host-immune responses to sepsis, there has been no translation to new therapies over the last two decades^12^.

**Figure 2.**
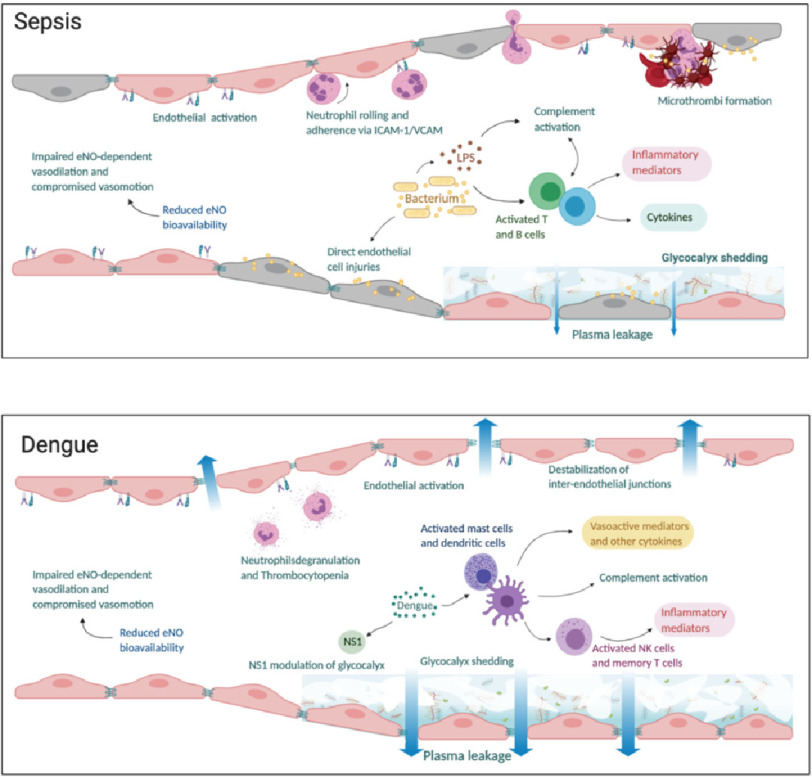
The microcirculation appearance in (a) sepsis and (b) dengue. In sepsis, there is inflammation and direct injury of endothelial cells by bacterial proteins and toxins. Endothelial activation leads to neutrophil chemotaxis and adherence to intercellular adhesion molecule- 1 (ICAM-1) and vascular adhesion molecule (VCAM) expressed on endothelial cells. Concurrently, there is activation of complement, T and B immune cells leading to release of inflammatory mediators and cytokines which cause further inflammation and glycocalyx damage. Concurrently, the coagulation cascade is activated, and anticoagulant/fibrinolysis pathways are impaired leading to formation of microthrombi and compromised microvascular flow. In dengue, viral non-structural 1 (NS1) protein triggers hyperpermeability and directly alters the endothelial layer function through the activation of several enzymes responsible for glycocalyx degradation. Activation of immune cells leads to complement activation and release of inflammatory mediators. There is impaired endothelial nitric oxide (eNO) dependent vasodilation due to reduced eNO bioavailability leading to compromised microvascular flow. Plasma leakage in both sepsis and dengue occur as a result of glycocalyx shedding and disruption of tight and adherens junctions between endothelial cells.

Dengue is an arboviral infectious disease caused by a single-stranded Flavivirus, the dengue virus (DENV) transmitted by *Aedes* mosquito vectors^15^. Dengue incidence continues to increase globally, driven in part by globalization, climate change and a lack of effective vaccines or prevention strategies^16,17^. There is a high disease burden in Southeast Asia, Asia, South America and emerging outbreaks throughout Africa^18^.

In both septic shock and dengue shock, the common hallmarks of microvascular dysfunction are endothelial cell activation and glycocalyx degradation leading to plasma leak through a disrupted endothelial barrier, together with reduced nitric oxide bioavailability leading to impaired microvascular tone^19–22^. Figure 2 shows the microcirculation in sepsis and in dengue. Despite similarity of endothelial dysfunction, the responses to fluid therapy in septic and dengue shock are very different. Fluid boluses have been shown to lead to clinical improvement in dengue shock^23^ but worsening outcomes in septic shock^24–27^, albeit fluid boluses in dengue shock tend to be of smaller volume.

While the final common pathway of glycocalyx shedding and microvascular dysfunction is similar in septic and dengue shock, the magnitude of the plasma leak appears to be greater in dengue infections^28^.

There are several similarities in microvascular dysfunction in septic and dengue shock, yet certain differences are also emerging, including the degree and specific components of glycocalyx disruption. Understanding these pathophysiological mechanisms may lead to improved management in the form of tailored fluid resuscitation and targeted therapeutics.

### Measuring microcirculatory dysfunction

No single measurement offers comprehensive evaluation of microvascular function, but combinations of the following techniques can provide insight into the nature of microcirculatory disturbance in septic shock and dengue shock.

Previous methods to quantify perfusion of the microcirculation *in vivo* used invasive techniques such as intravital microscopy trans-illumination in experimental animals^29^ or human nail-fold beds^30,31^ and non-invasive laser speckle and laser Doppler tracking whereby a monochromatic laser light is scattered by red blood cells moving through the microcirculation^32,33^. However, heterogeneity of microvascular beds in different organs, cost, complexity and invasiveness of assessment precluded routine assessment of the microcirculation in clinical settings using these techniques.

**Figure 3.**
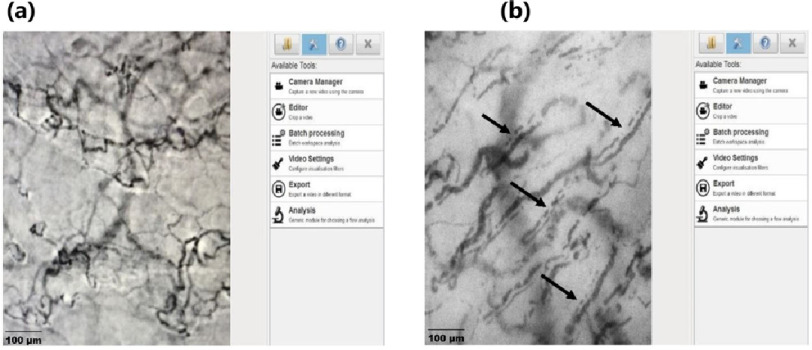
Sublingual microcirculation in (a) normal (b) in septic shock. The micro-vessels in septic shock have a comparatively larger diameter and do not run the entire course resulting in a dotted-line appearance characteristic of impaired microvascular flow in sepsis shown by the arrows in (b). *Figures courtesy Critical Care Research Group (CCRG) laboratory, QLD, Australia*.

In light of these shortcomings, videomicroscopic techniques based on orthogonal polarisation spectroscopy are increasingly used for direct, non-invasive imaging of the microcirculation. Real-time visualisation of the microcirculation is now possible with dark-field imaging technology. Using side-stream dark-field (SDF) or incident dark-field (IDF) videomicroscopy, it is possible to visualize red blood cells flowing in the microcirculation through mucus membranes^34^. Hand-held videomicroscopes have made it possible to image the sublingual microcirculation in real-time during critical illness, to measure the proportion of perfused micro-vessels, assess heterogeneity in flow within the microvascular bed, and estimate the depth of the endothelial glycocalyx as a proxy for degradation. Although SDF/IDF videomicroscopy is predominantly a research tool at present, recent advances to automate analysis may make it feasible to evaluate microcirculatory dysfunction and measure response to therapies in the clinical setting^35^. Figure 3 shows a comparison normal and septic sublingual microcirculation.

Non-invasive assessment of endothelial nitric oxide dependent vasodilation can be performed using real-time techniques such as brachial artery flow mediated dilatation, and EndoPAT - an automated, user-independent technique^22,36^.

Biomarkers of glycocalyx degradation and endothelial activation can be measured in circulating plasma; such markers provide an indirect measurement of the mechanism, timing, and extent of glycocalyx and endothelial barrier disruption. In addition, measuring urinary clearance of heparan sulphate can be a useful, non-invasive, indicator of glycocalyx turnover^28,37^.

### Microvascular dysfunction in sepsis

Sepsis is fundamentally an inflammatory disease initiated by host recognition of infection-derived antigen proteins^38^; however, this host-response to the infection is dysregulated leading, in some cases, to life-threatening organ dysfunction^1^. Septic shock results from untreated or partially treated sepsis progressing to cause circulatory, cellular and metabolic abnormalities associated with a higher risk of mortality^1^.

Direct injury of the microvascular endothelium by release of endotoxins, myocardial depressant factors as well as a physiologic cascade of pro-inflammatory cytokines cause dysfunction of the circulatory system in septic shock; these activate a cascade leading to formation of micro-thrombi and impairment of micro-vessel perfusion, endothelial cell activation, disruption of the endothelial-glycocalyx layer and leakage of plasma into the extravascular space. This leads to a drop in the circulating intravascular volume, manifesting clinically as low blood pressure.

Compounding this is a state of reduced nitric oxide bioavailability, resulting in impaired endothelial nitric oxide dependent vasodilation and compromised vasomotion. Nitric oxide is produced by catalytic oxidation of L-arginine by nitric oxide synthase (NOS) enzyme^39^. Increased arginase activity^40^ leading to reduced L-arginine is also likely contributory to this hypotensive state in sepsis^41^.

The cornerstone of resuscitation in sepsis has therefore been fluid administration to restore the circulating volume, with the addition of vasopressors to restore microvascular tone and improve myocardial contractility, if necessary. The use of fluid boluses for resuscitation in septic shock has, however, been challenged by clinical and pre-clinical trials. In 2011, the Fluid Expansion As Supportive Therapy (FEAST) trial, demonstrated a 45% increase in the relative risk of mortality with fluid bolus treatment compared to non-bolus controls (95% CI [1.13–1.86]; *p* = 0.003)^24^ with excess mortality following fluid bolus therapy being attributed to fatal cardiovascular collapse^42^. In 2017, Andrews *et al* reported a 46% higher in-hospital mortality among predominantly HIV-positive adults with sepsis and hypotension receiving protocolised early intravenous fluids and vasopressors compared to usual care (95% CI [1.04–2.05]; *P* = 0.03)^43^. A recent pre-clinical trial revealed a paradoxical increase in vasopressor requirement, a rise in cardiac troponin I and atrial natriuretic peptide as well as increased shedding of hyaluronan, a component of the endothelial-glycocalyx, following fluid bolus resuscitation of endotoxaemic shock compared to non-bolus control^44^.

Improvements in systemic macro-haemodynamic parameters after resuscitation do not necessarily lead to an improvement in microcirculatory parameters; this ‘loss of haemodynamic coherence’ is an independent predictor of adverse patient outcome^45^. Indeed, after macrocirculatory optimisation, attempts to directly target the microcirculation with inhaled nitric oxide did not augment microcirculatory perfusion, enhance lactate clearance or reduce organ dysfunction in patients with septic shock^46,47^. Given these findings, further work is needed to find the best strategies to optimise microcirculatory function in septic shock.

### Microvascular dysfunction in severe dengue

**Figure 4.**
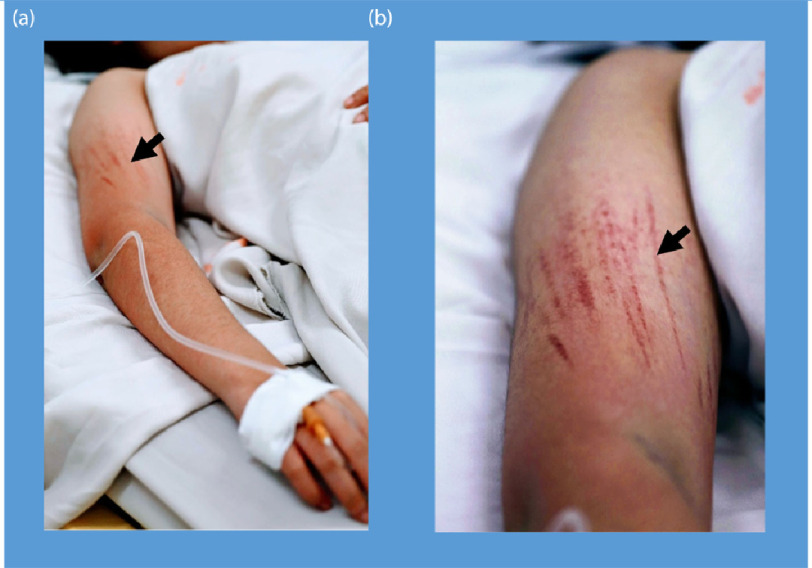
Bleeding manifestations in dengue shock syndrome showing the appearance of linear petechiae after blood pressure cuff inflation.

Dengue is a viral illness with a broad spectrum of clinical phenotypes. While the majority of infections are mild and self-limiting, a small proportion of patients develop dengue shock syndrome due to an increase in vascular permeability, profound plasma leakage and subsequent hypovolaemia^48,49^. Figure 4 shows bleeding manifestations seen clinically in dengue patients.

Understanding of the mechanisms underlying microcirculatory injury in dengue is still incomplete. The dengue non-structural 1 (NS1) protein, a viral glycoprotein secreted by infected cells, has been shown to cause an increase in endothelial permeability leading to vascular leakage^50^. Of note, recent findings from Glasner *et al* have shown that NS1-mediated vascular leak depends on the integrity of endothelial glycocalyx components, both *in vitro* and *in vivo*, rather than inflammatory cytokines^51^. The Dengue-NS1 protein induces expression of several enzymes which degrade glycocalyx components, including heparanases and sialidases^52^.

In parallel, the host immune response to dengue infection is widely recognised to play a key role in microcirculatory injury^53,54,55^; excessive pro-inflammatory cytokines, particularly tumour necrosis factor-alpha and other vasoactive mediators, also cause tissue injury resulting in an increase in capillary permeability^56^.

SDF videomicroscopy has shown reduced perfusion of the sublingual microcirculation in patients with plasma leakage during the critical phase of dengue infection, together with a reduction in glycocalyx depth and elevated plasma levels of glycocalyx components, both most marked in patients with the most severe plasma leak^57^. Indeed, plasma syndecan-1 levels are several-fold higher in patients with dengue shock versus septic shock, which suggests a key difference in the magnitude or mechanisms underlying microcirculatory damage between the syndromes^4,58,59^. In vitro studies have also demonstrated rearrangement of intra-cellular actin filaments, enhanced endothelial cytoskeleton contractility, destabilisation of inter-endothelial adherens junctions and decreased expression of tight junction protein ZO-1^60,61^; all of these changes likely contribute to plasma leakage. EndoPAT studies have shown impaired endothelial NO-dependent vasodilation, which might be partly explained by both glycocalyx breakdown and reduced NO bioavailability due to a decrease in L-arginine and increase in arginase during dengue infection^22^. However, the small proportion of patients who require vasopressors to treat dengue shock compared to septic shock suggests that impairment in microvascular tone is more marked in the latter syndrome^62^.

At present, the cornerstone of dengue shock management is judicious resuscitation with intravenous fluid, with the occasional need for vasopressors, until the critical phase of plasma leakage ends. Notably, only a minority of children tend to require vasopressor support in dengue shock syndrome, with more adults needing haemodynamic support^63^. Based on evidence from trials of resuscitation with different types of intravenous fluid, the WHO guidelines recommend the initial use of crystalloid solutions, followed by colloid solutions for patients with unresponsive shock^18,23^. There is a paucity of evidence from randomised controlled trials on how rate and volume of fluid resuscitation affects outcomes in dengue shock. Learning from research on septic shock, studies are needed to explore whether optimisation of macrocirculatory parameters improves microcirculatory function, or whether current fluid management strategies may be detrimental to an already injured glycocalyx layer in dengue shock.

### Summary and future directions

Impairment in perfusion, tone and barrier-function of the microcirculation is central to the pathogenesis of both septic shock and dengue shock; but it is clear from in vitro and clinical studies that the nature and extent of dysfunction differs between the syndromes. These differences may impact the likelihood of responding to treatment; volume expansion, vasopressors to enhance microvascular tone or therapeutics aimed at restoring the glycocalyx layer and endothelial barrier may be differentially effective based on the underlying pathology.

Studies in other conditions investigating different resuscitation fluids on the integrity of the glycocalyx and endothelial function have shown a beneficial effect of fresh frozen plasma (FFP) on glycocalyx restoration and improved vascular permeability^64,65^. The exact components of FFP and the potential mechanisms responsible for this glycocalyx repair remain to be defined, but preformed SDC1 in the FFP is thought to restore the layer resulting in improved barrier and endothelial cell functions^66^. In addition, synthetic colloids are thought to transiently restore the capillary permeability barrier properties by the incorporation of small dextran molecules into the glycocalyx layer. In an animal model of ischemic-reperfusion injury, infusion with hydroxyethyl starch (HES) reduced the net coronary fluid filtration^67^.

Findings from this study suggested that it may be beneficial to give low dose continuous colloid infusion rather than repeated larger colloid boluses in infection related shock syndrome, which could not only reduce the plasma leakage but also allow for lower total intravenous fluid volumes to be infused, potentially avoiding complications associated with fluid overload.

Similarly, Muller *et al* reported decreased endothelial damage following resuscitation with HES compared to Ringer’s lactate in a clinical sub-study of the Scandinavian Starch for Severe Sepsis/Septic shock (6S) randomized clinical trial (RCT)^68^. However, overall mortality was higher and there was an increased requirement for dialysis in septic patients who received starch-based resuscitation in the 6S RCT^69^. This has brought to question the relevance of biomarkers of endothelial damage as surrogates of organ damage and clinical outcomes. The Australian and New Zealand Intensive Care Society Clinical Trial Group (ANZICS-CTG) did not show a difference in the 90-day mortality between HES- and saline-resuscitated critically ill patients, however there was an increased need for dialysis with HES administration^70^. Findings from these RCTs have resulted in limited use of starch-based solutions for resuscitation in critical illness.

Intravenous fluid resuscitation is likely to remain a cornerstone of management for both syndromes; however, more work is needed to evaluate the impact of liberal versus restrictive fluid resuscitation, optimal choice and rate of fluid administration to prevent further damage to the ailing glycocalyx, and non-invasive methods to assess changes in microvascular function in response to treatment. Aside from using glycocalyx protective or restorative intravenous fluids, interest is emerging in the glycocalyx as a target for novel host-directed therapeutic agents in infectious shock. Progress in this field has thus far been largely confined to pre-clinical studies in animals with induced sepsis. Some strategies which hold potential for translation to humans include; reducing glycocalyx breakdown by downregulating the matrix metalloproteinases responsible for SDC1 shedding (sphingosine-1-phosphate analogues^71,72^), inhibiting heparanase activity (modified heparins, Sulodexide^73^, Tie-2 agonists^74,75^), or accelerating glycocalyx restitution by activating enzymes such as exostosin-1, involved in the heparan sulphate synthesis pathway (fibroblast growth factor^76^). Progress in screening such glycocalyx-directed therapeutics for dengue has been impeded thus far due to a lack of adequate pre-clinical model for dengue-induced plasma leakage.

Although ultimately the best treatment strategies might be different, sharing lessons learned from studies on the microcirculation in septic shock and dengue shock may help to advance management and improve patient outcomes in a bi-directional manner.

## Funding

No funding was received for preparation of this manuscript.

AMB is funded through a Wellcome Trust PhD Fellowship Award [203905/Z/16/Z].

NGO is funded through the DELTAS Africa Initiative [DEL-15-003]. The DELTAS Africa Initiative is an independent funding scheme of the African Academy of Sciences (AAS)’s Alliance for Accelerating Excellence in Science in Africa (AESA) and supported by the New Partnership for Africa’s Development Planning and Coordinating Agency (NEPAD Agency) with funding from the Wellcome Trust [107769/Z/10/Z] and the UK government.

The views expressed in this publication are those of the author(s) and not necessarily those of AAS, NEPAD Agency, Wellcome Trust or the UK government.

## Declarations

SY receives consultancy fees from Janssen pharmaceuticals for dengue antiviral development and as a member of the ROCHE Advisory Board on Severe Dengue.

All other authors declare no conflicts.

## Authors contributions

AMB, HQC and NGO wrote the original draft of the manuscript. All authors reviewed, edited and approved submission of the manuscript.
